# Ultrasonographic Diagnosis of Fetal Hypospadias

**DOI:** 10.3390/diagnostics12040774

**Published:** 2022-03-22

**Authors:** Kim-Seng Law

**Affiliations:** 1 Department of Obstetrics and Gynecology, Tung’s Taichung MetroHarbor Hospital, Taichung 435, Taiwan; kimsenglaw@gmail.com; Tel.: +886-916120758; 2 Department of Post-Baccalaureate Medicine, College of Medicine, National Chung Hsing University, Taichung 402, Taiwan

**Keywords:** color Doppler, hypospadias, ultrasound

## Abstract

Fetal hypospadias should be consider in a male fetus with a shortened penis, blunt bulbous tips, ventrally curved shaft with or without chordee and a typical fan shape stream of urinary jet under color Doppler under prenatal ultrasound examination. The more severe form is usually accompanied by other congenital abnormalities.

## 1. Introduction

Hypospadias is characterized by an abnormal placement of the external urethral meatus in male infants usually accompanied by ventrally bent penile shaft with dorsal hood of the prepuce.

**Figure 1:**
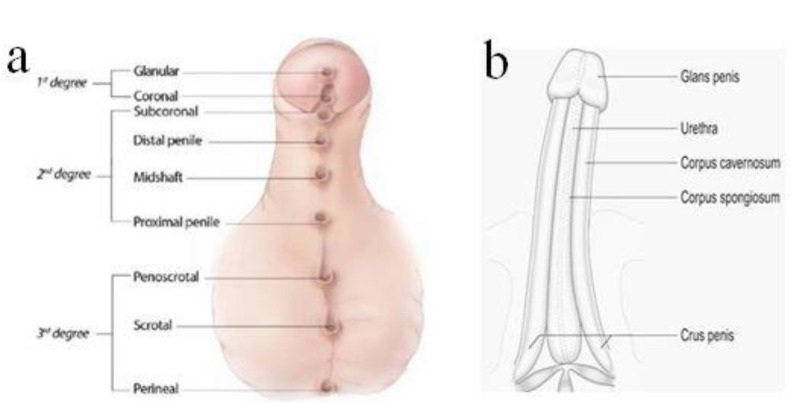
Classification of hypospadias according to the opening of meatus (**a**). Normal penile shaft (**b**).

Hypospadias is classified by severity, depending on the location of the meatus: first-degree hypospadias includes the more distal forms, glanular and coronal; second-degree hypospadias includes subcoronal and penile shaft hypospadias; and third degree—the most severe—includes scrotal and perineal hypospadias (Figure 1a) [1,2].

Although positive predictive value of prenatal diagnosis of hypospadias is reported to be 72%, it might be difficult, especially in those with first degree with meatus opening at the glanular or coronal, which need a color Doppler to detect the specific jet of the urinary stream [3].

## 2. Case Reports

### 2.1. Case 1

**Figure 2:**
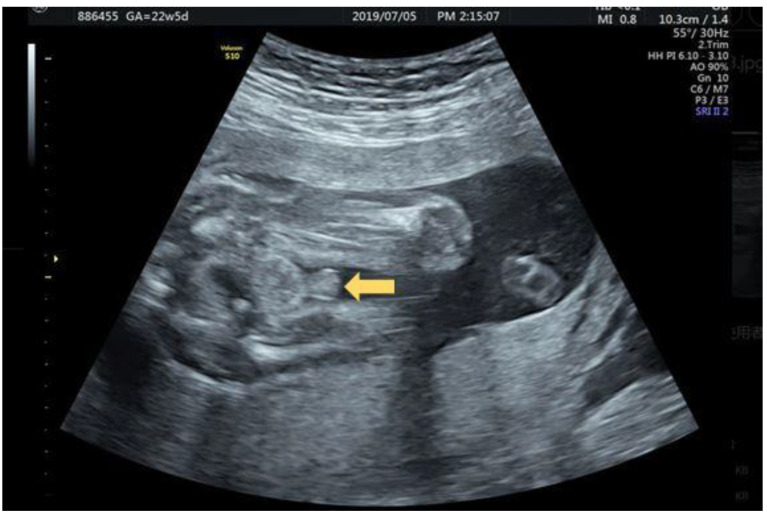
32 year old G3P1A1 GA 22 + 5 weeks with “blunt” instead of taper “pointed” penis tip (arrow).

**Figure 3:**
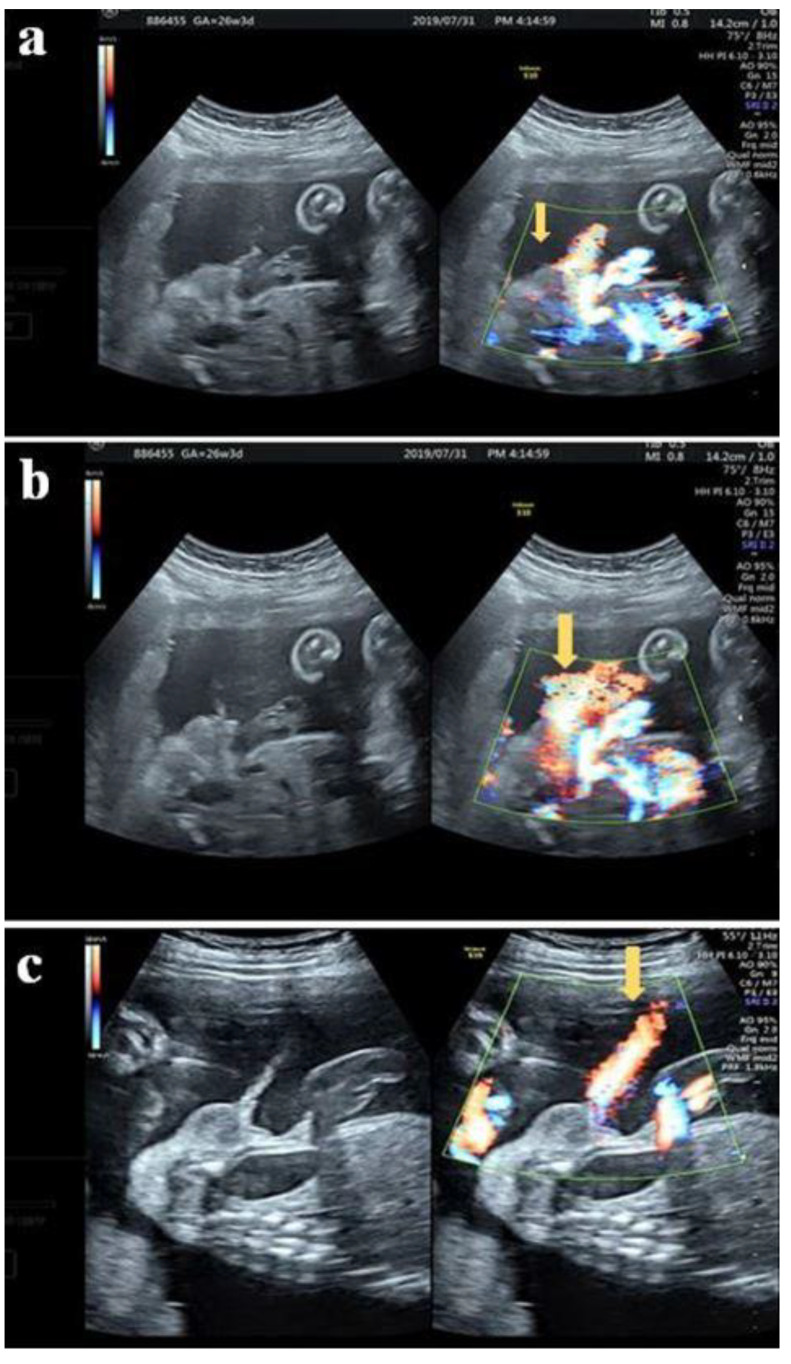
(**a**) The same patient at 26 + 3 weeks with stream of “coarse” urinary jets seen initially under color Doppler (arrow) (**b**) Typical fanning stream of the urinary jet revealed follows the initiated “coarse” jet of urinary stream (arrow) (**c**) Normal voiding under color Doppler with “linear” jet of stream (arrow).

A 32-year-old female G3P1A1 with normal antenatal screening and normal Quad test except with a blunt bulbous penile tip (Figure 2) noted under second trimester ultrasound, raising the suspicious of isolated hypospadias. The same patient was examined at 26 + 3 weeks gestational age with jet of “coarse “ urinary stream (Figure 3a) follow by typical fanning of urinary stream jet (Figure 3b) after external provocation, confirming a first degree (distal) hypospadias.

**Figure 4:**
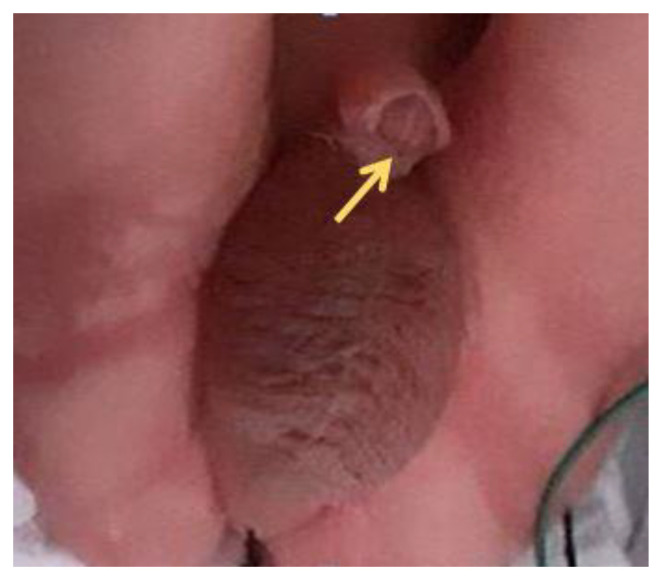
A ventrally bent penis with first degree hypospadias.

The patient developed preeclampsia at 34 + 5 weeks with cesarean delivery of a male baby weighing 1944 g. A first degree hypospadias was confirmed postnatal (Figure 4).

### 2.2. Case 2

**Figure 5:**
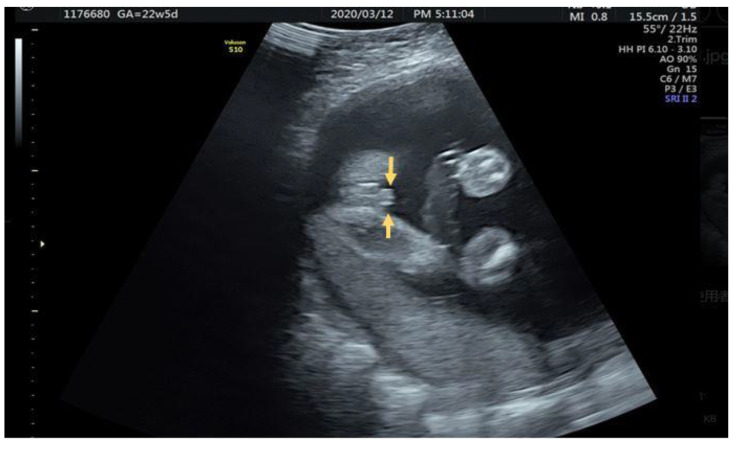
Parallel hyperechoic lateral prepuce fold alongside penis (arrow).

A 25-year-old female G1P0 with normal antenatal examination and NIPS with short penis shaft and blunt tip accompanied with two lateral hyperechogenic lateral fold of prepuce was noted at 22 + 5 weeks (Figure 5).

**Figure 6:**
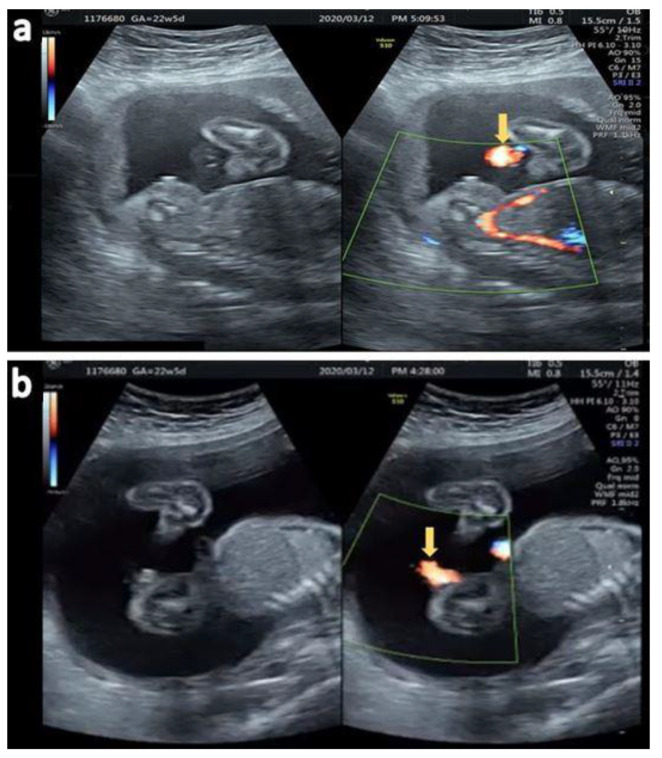
(**a**) Fanning of the urinary stream jet proximal to the perineum (arrow) (**b**) Sagittal view of the ventral jet of urinary stream under color Doppler (arrow).

Axial view (Figure 6a) and sagittal view (Figure 6b) of the urinary jet of stream under color Doppler confirming a third degree hypospadias. She visited another hospital and was lost in follow-up.

### 2.3. Case 3

**Figure 7:**
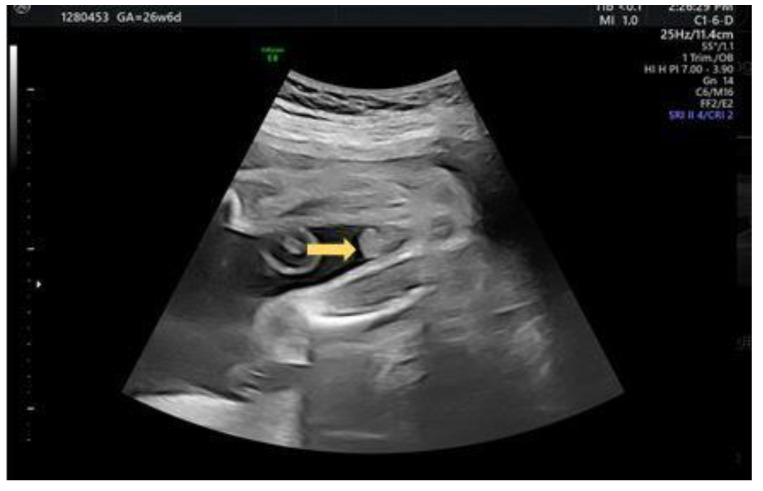
Axial view of a blunt bulbous penis tip (arrow).

**Figure 8:**
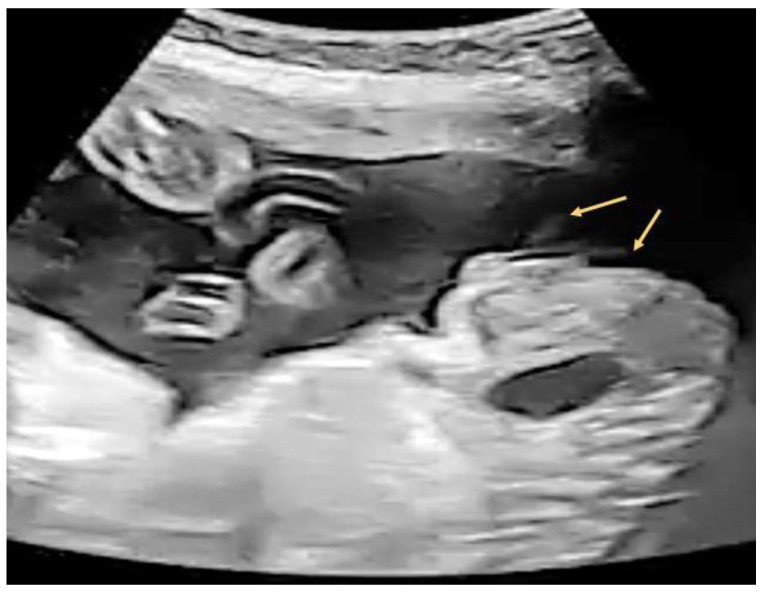
Split jet of urinary stream (arrows) seen after external provocation under gray scale.

A 29-year-old female G4P2A1 pregnant at 26 + 2 weeks with normal antenatal examination and Quad test with blunt penile tip noted (Figure 7). “Split” form of urinary jet was seen under gray scale (Figure 8).

**Figure 9:**
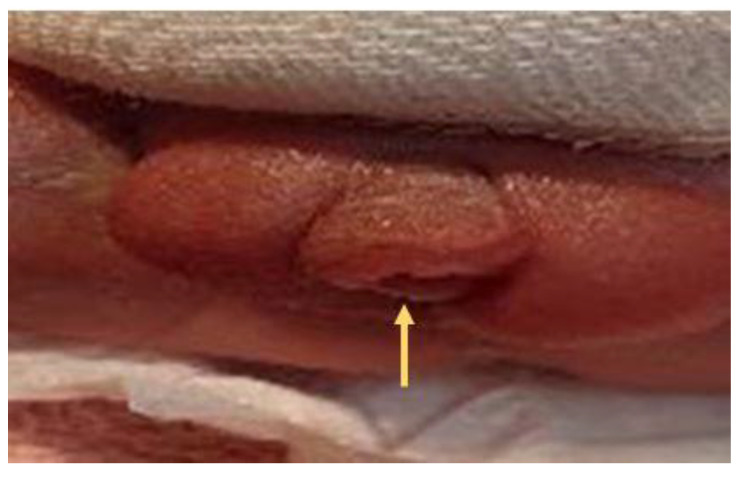
A second degree (proximal penis) hypospadias was noted with meatus (arrow) opening at the proximal shaft of penis.

She gave birth to a 1232 g baby at gestational age of 32 + 6 weeks due to premature rupture of membranes through cesarean section with a second degree hypospadias noted postnatal (Figure 9).

### 2.4. Case 4

**Figure 10:**
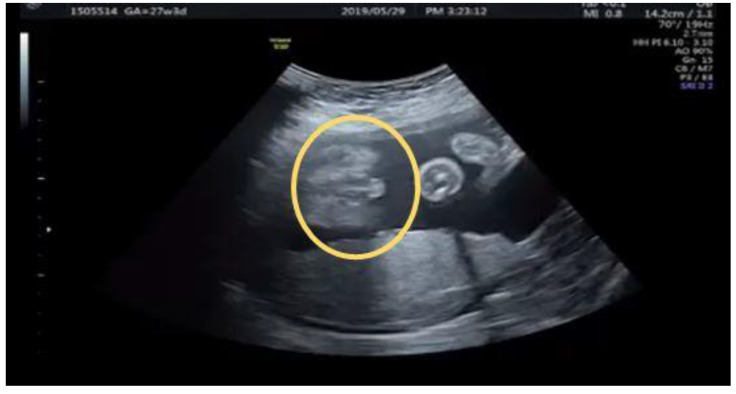
Third degree hypospadias with typical “Tulip” signs (circular).

**Figure 11:**
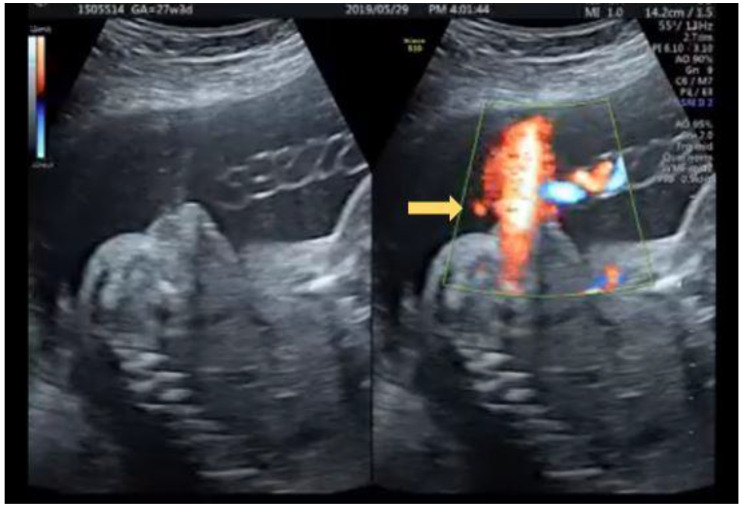
Fanning of urinary stream originated proximal to perineum under color Doppler (arrow).

A 26-year-old female G1P0 pregnant at 27 + 3 gestational age was noted to have “tulip” signs over external genitalia resemblance a third degree hypospadias (Figure 10). Sagittal view after external provocation with jet of fanning urinary stream originated directly from perineum was noted (Figure 11). Coarctation of aorta and pulmonary stenosis was also noted in this fetus. Normal karyotyping with 46XY was found.

**Figure 12:**
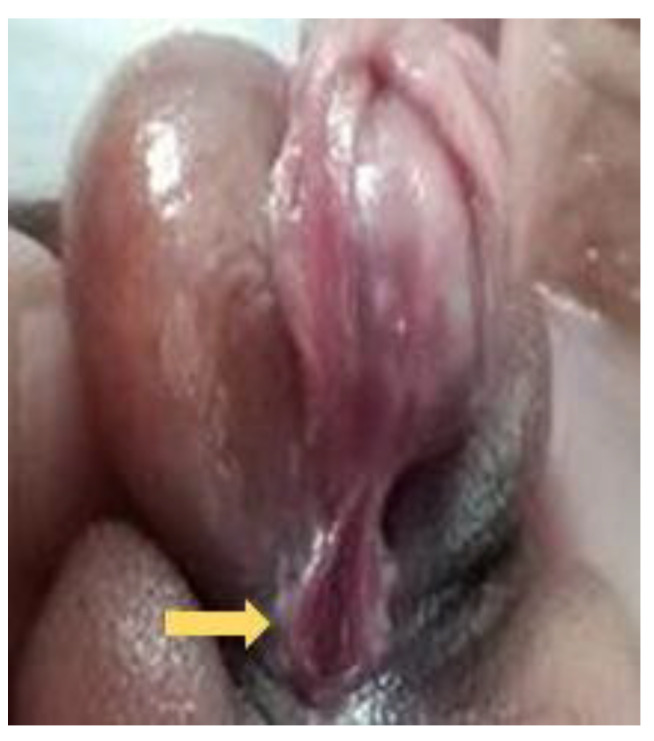
Third degree (severe) hypospadias with meatus opening over penoscrotal area (arrow).

A male baby was delivered through cesarean section weighing 2624 g, and third degree hypospadias was confirmed postnatal (Figure 12).

## 3. Discussion

Hypospadias is the second most common urinary congenital anomalies secondary to undescended testis with a prevalence rate of 18.6/10,000 in Europe, 34.2/10,000 in North America and 0.6–69/10,000 in Asia [4].

Hypospadias is often an isolated (>80%), non-syndromic anomaly. However, the proportion of isolated defects decreases with increasing severity (second and third degree) of hypospadias.

Hypospadias can also be associated with underlying genetic syndromes, such as Smith-Lemli-Opitz, Wolf-Hirschhorn, Opitz G/BBB, Schilbach-Rott, hand-foot-genital, trisomies 13 and 18, triploidy, and many others. Androgen insensitivity, adrenal hyperplasia have also been reported in conjunction with hypospadias [4,5,6,7].

Other risk factors for hypospadias include advanced maternal age (>35 years), maternal hypertension, oligohydramnios and small for gestational age and environmental exposure to certain chemicals (endocrine disruptors).

Prenatal diagnosis is generally made in the third trimester in early reports with the advance of sonographic technique, it is possible to confirming the diagnosis during the early second trimester with the advance of ultrasound technique. Most prenatally diagnosed cases have been attributed to distal hypospadias with ultrasound findings of anomalous distal morphology of the penis, small lateral folds, small penis with ventral incurvation or an anomalous urinary stream [4,5,8].

The normal penile shaft has a smooth tapering pointed tip appearance.

In hypospadias, the tip of the penis is blunted and bulbous with two echogenic lines corresponding to small lateral folds belonging to the dermal remains of the prepuce (dorsal hood) these are usually the first signs for a suspicious hypospadias.

Slight ventral or lateral curvature of the penile shaft resulting from tethering fibrous band, the chordee should also raise the suspicious of a hypospadias.

“Tulip sign” represents the severe curvature of the penis in association with penoscrotal transposition of a bifid scrotum. Thus, this sign indicates the most severe form of hypospadias (third degree).

Observing the flow of urine can aid in the diagnosis and in establishing the degree/severity of the condition. Fetal micturition occurring proximal to the glans may be demonstrated on gray scale or color Doppler images. The stream is often fan-shaped and not linear with ventral deflection of the urinary stream jet always observed as seen in our cases. Most antenatal screening of fetal hypospadias could be carried out after 20 weeks of gestational age with color Doppler showing the fanning of urinary stream as a confirmatory tool in fetus with short and blunt penis. Additional searching for other anomalies and karyotyping should be consider for further evaluation.
